# A Matched Molecular and Clinical Analysis of the Epithelioid Haemangioendothelioma Cohort in the Stafford Fox Rare Cancer Program and Contextual Literature Review

**DOI:** 10.3390/cancers15174378

**Published:** 2023-09-01

**Authors:** Arwa Abdelmogod, Lia Papadopoulos, Stephen Riordan, Melvin Wong, Martin Weltman, Ratana Lim, Christopher McEvoy, Andrew Fellowes, Stephen Fox, Justin Bedő, Jocelyn Penington, Kym Pham, Oliver Hofmann, Joseph H. A. Vissers, Sean Grimmond, Gayanie Ratnayake, Michael Christie, Catherine Mitchell, William K. Murray, Kelly McClymont, Peter Luk, Anthony T. Papenfuss, Damien Kee, Clare L. Scott, David Goldstein, Holly E. Barker

**Affiliations:** 1 Limestone Coast Local Health Network, Flinders University, Bedford Park, SA 5042, Australia; abde0058@flinders.edu.au; 2 The Walter and Eliza Hall Institute of Medical Research, Parkville, VIC 3052, Australia; papadopoulos.l@wehi.edu.au (L.P.); lim.ra@wehi.edu.au (R.L.); bedo.j@wehi.edu.au (J.B.); penington.j@wehi.edu.au (J.P.); papenfuss@wehi.edu.au (A.T.P.); kee.d@wehi.edu.au (D.K.); scottc@wehi.edu.au (C.L.S.); 3 The Australian Rare Cancer Portal, BioGrid, Parkville, VIC 3051, Australia; david.goldstein@health.nsw.gov.au; 4 Eastern Health Clinical School, Monash University, Box Hill, VIC 3128, Australia; 5 Prince of Wales Clinical School, University of NSW, Randwick, NSW 2031, Australia; stephen.riordan@health.nsw.gov.au; 6 Gastrointestinal and Liver Unit, Prince of Wales Hospital, Randwick, NSW 2031, Australia; 7 Radiology Department, Prince of Wales Hospital, Randwick, NSW 2031, Australia; melvin.wong@health.nsw.gov.au; 8 Department of Gastroenterology, Nepean Hospital, Kingswood, NSW 2747, Australia; martin.weltman@health.nsw.gov.au; 9 Peter MacCallum Cancer Centre, Melbourne, VIC 3000, Australia; christopher.mcevoy@petermac.org (C.M.); andrew.fellowes@petermac.org (A.F.);; 10 Centre for Cancer Research and Department of Clinical Pathology, University of Melbourne, Melbourne, VIC 3010, Australia; kym.phamstewart@unimelb.edu.au (K.P.); oliver.hofmann@unimelb.edu.au (O.H.); joseph.vissers@unimelb.edu.au (J.H.A.V.); sean.grimmond@unimelb.edu.au (S.G.); 11 The Royal Womens’ Hospital, Parkville, VIC 3052, Australia; gayanie.ratnayake@rch.org.au; 12 The Royal Melbourne Hospital, Parkville, VIC 3052, Australia; michael.christie@mh.org.au; 13 Department of Pathology, Peter MacCallum Cancer Centre, Melbourne, VIC 3000, Australia; catherine.mitchell@petermac.org (C.M.); bill.murray@petermac.org (W.K.M.); 14 Sullivan Nicolaides Pathology, Brisbane, QLD 4000, Australia; kelly_mcclymont@snp.com.au; 15 Royal Prince Alfred Hospital, Camperdown, NSW 2050, Australia; peter.luk@health.nsw.gov.au; 16 Sir Peter MacCallum Cancer Centre, Department of Oncology, University of Melbourne, Parkville, VIC 3000, Australia; 17 Austin Health, Heidelberg, VIC 3084, Australia; 18 Department of Medical Biology, University of Melbourne, Melbourne, VIC 3010, Australia; 19 Department of Obstetrics and Gynaecology, University of Melbourne, Parkville, VIC 3010, Australia; 20 Nelune Center, Prince of Wales Hospital, Randwick, NSW 2031, Australia

**Keywords:** epithelioid haemangioendothelioma, histopathology, genomics, rare cancer, SFRCP, ARC Portal, fusion genes, biomarker, YAP, TAZ

## Abstract

**Simple Summary:**

Epithelioid haemangioendothelioma (EHE) is an ultra-rare malignant vascular tumour with a prevalence of 1 per 1,000,000. It develops from endothelial cells, which are the cells that line all blood vessels in the body. Therefore, it typically expresses endothelial cell markers. It can also be identified through analysis of the genes. Two genes, *WWTR1* and *CAMTA1,* are broken and fused together in 90% of cases. Alternatively, in approximately 10% of cases, the genes that are broken and fused together are *YAP1* and *TFE3*. We analysed an Australian cohort of EHE patients to look for associations between genetic changes and clinical characteristics. EHE cases are typically refractory to therapies, and no anticancer agents are reimbursed for EHE in Australia; therefore, this detailed study of EHE adds important information to advance our understanding and help aid the design of treatment regimens in the future.

**Abstract:**

Background: Epithelioid haemangioendothelioma (EHE) is an ultra-rare malignant vascular tumour with a prevalence of 1 per 1,000,000. It is typically molecularly characterised by a *WWTR1::CAMTA1* gene fusion in approximately 90% of cases, or a *YAP1::TFE3* gene fusion in approximately 10% of cases. EHE cases are typically refractory to therapies, and no anticancer agents are reimbursed for EHE in Australia. Methods: We report a cohort of nine EHE cases with comprehensive histologic and molecular profiling from the Walter and Eliza Hall Institute of Medical Research Stafford Fox Rare Cancer Program (WEHI-SFRCP) collated via nation-wide referral to the Australian Rare Cancer (ARC) Portal. The diagnoses of EHE were confirmed by histopathological and immunohistochemical (IHC) examination. Molecular profiling was performed using the TruSight Oncology 500 assay, the TruSight RNA fusion panel, whole genome sequencing (WGS), or whole exome sequencing (WES). Results: Molecular analysis of RNA, DNA or both was possible in seven of nine cases. The *WWTR1::CAMTA1* fusion was identified in five cases. The *YAP1::TFE3* fusion was identified in one case, demonstrating unique morphology compared to cases with the more common *WWTR1::CAMTA1* fusion. All tumours expressed typical endothelial markers CD31, ERG, and CD34 and were negative for pan-cytokeratin. Cases with a *WWTR1::CAMTA1* fusion displayed high expression of CAMTA1 and the single case with a *YAP1::TFE3* fusion displayed high expression of TFE3. Survival was highly variable and unrelated to molecular profile. Conclusions: This cohort of EHE cases provides molecular and histopathological characterisation and matching clinical information that emphasises the molecular patterns and variable clinical outcomes and adds to our knowledge of this ultra-rare cancer. Such information from multiple studies will advance our understanding, potentially improving treatment options.

## 1. Introduction

Epithelioid haemangioendothelioma (EHE) is a rare, low-to-intermediate grade malignant tumour of vascular origin that may develop in the extremities, soft tissue, lung, bone, and liver. The name was first coined in 1982 by Weiss and Enzinger due to its overlapping features between a haemangioma and angiosarcoma. It is a very rare tumour with a prevalence of 1 per 1,000,000 in the general population [1].

The World Health Organisation (WHO) has recommended that EHE be grouped with malignant tumours [2]. It has an unpredictable clinical behaviour ranging from indolent to aggressively malignant, with a mean survival of 4.6 years, ranging from 6 months to 24 years [2]. Risk stratification models have been proposed to identify lesions at high risk for tumour progression, with tumour cellularity, tumour size, high mitotic figures, and a Ki67 score greater than 10% being the most significant indicators for poor prognosis [3,4]. 

EHE has no standard treatment regimen, and very few therapeutic options are available. Historically, liver resection and transplantation has been the only curative option for patients with hepatic EHE because of tumour multifocality, but is associated with variable outcomes [5]. However, an expanding oncology therapeutic landscape and advances in genomic tumour analysis may help direct the choice of potentially active treatments, such as those targeting vascular endothelial growth factor (VEGF) or immunotherapy. Herein, we review the current literature for EHE and report on a cohort of hepatic and extrahepatic EHE diagnoses based on histopathology and molecular studies. We aim to add to the current literature on this ultra-rare cancer so that we can better understand the behaviour and natural history of EHE in Australian patients, hopefully helping to better inform treatment-making decisions in the future.

## 2. Background Literature Review

### 2.1. Radiological Characterisation

Radiologic findings are often nonspecific and vary according to the site. Two characteristic computed tomography (CT) and magnetic resonance imaging (MRI) findings in hepatic EHE include the “lollipop sign”, in larger lesions (>5 cm) due to bridging vein thrombosis with a rounded “head” and a tapering “tail” [6], and the “target sign”, which is a lesion with a low intensity central area surrounded by a hyperintense rim, and is more likely to be found in smaller lesions (2–5 cm) [7]. Of note, benign-looking pulmonary calcification and hepatic capsular retractions are common findings in positron-emission topography (PET)/CT scans of pleural and hepatic EHE, respectively [8]. 

### 2.2. Histopathological Features

Morphologically, EHE is most often composed of epithelioid cells, organised in nests or cords, with eosinophilic to vacuolated cytoplasm, set in a distinctive myxohyaline stroma, and occasionally associated with haemorrhagic foci [9]. Blister cells with intraluminal erythrocytes, mild-to-moderate atypia, and a low rate of mitosis may also be present [10]. Immunohistochemical (IHC) stains show EHE consistently expresses endothelial cell markers (CD31, CD34 and ERG), and up to 40% of cases may be positive for cytokeratins [11]. EHE with a *YAP1::TFE3* fusion has several distinct morphologic features, including voluminous cytoplasm, well-formed vascular lumens, solid growth, and minimal or no stroma [10]. A minority of cases show atypical or malignant features, such as increased cytological atypia and increased mitotic activity with or without necrosis, and may have morphologic overlap with epithelioid angiosarcoma [12]. 

### 2.3. Molecular Characterisation

The WHO classification of sarcomas and the European Society of Medical Oncology (ESMO) consensus have set two gene translocations, both involving the Hippo pathway, as disease-defining fusion genes for EHE [13,14]. The Hippo pathway is involved in normal development, cell growth and homeostasis, hence its dysfunction can lead to the development and progression of multiple cancers [15,16]. The pathognomonic *WWTR1::CAMTA1* fusion, which results from a t(1;3)(p36.3;q25) translocation, and the less common *YAP1::TFE3* fusion are considered diagnostic fusion genes [13,14,17]. Most cases of EHE are characterised by *WWTR1::CAMTA1* (90%) or *YAP1::TFE3* (10%) gene fusions [14]. 

Transcriptional co-activator with PDZ-binding motif (TAZ), encoded by the *WWTR1* gene, regulates the activity of various transcription factors, including the Transcriptional enhancer associated domain (TEAD) transcription factors, which play roles in cell proliferation and apoptosis [18]. Nuclear translocation of TAZ is inhibited by upstream proteins in the Hippo pathway [18]. The fusion between TAZ and CAMTA1 results in constitutive activation of TAZ, hyperactivation of TEAD-based transcriptional programs, and upregulated cellular proliferation [19,20] which may be inhibited with drugs that modulate TEAD activity [21].

YAP1 is a transcriptional coactivator, also controlled by the Hippo signalling pathway, which also interacts with transcription factors, such as TEADs, to promote growth and inhibit apoptosis [18]. TFE3 belongs to the MiTF/TFE family of basic helix-loop-helix (bHLH) transcription factors and regulates lysosomal biogenesis and energy homeostasis [22]. The YAP1::TFE3 fusion protein also drives hyperactivation of TEAD-based transcription programs [19]. 

These different fusion genes give rise to diverse biologic behaviour. Some propose that YAP1-TFE3 fusion tumours should be classified as a distinct entity given their unique clinical and histopathologic characteristics in comparison to conventional EHE [9,23]. In addition, YAP1::TFE3 fused EHE tends to arise in younger patients and has a more favourable outcome than EHE characterised by TAZ::CAMTA1 fusions, with the 5-year overall survival being 86% versus 59%, respectively [23,24].

Next-generation DNA sequencing (IMPACT) and targeted RNA sequencing (Archer FusionPlex Custom Solid Panel) on cohorts of 18 and 49 patients have uncovered additional drivers; at least 22% of cases had alterations in cell cycle and epigenetic pathways, and/or loss-of-function alterations in the DNA damage response pathways. These included pathogenic variants in *XRCC1/2*, *ERCC1*, *RB1*, *APC*, *FANCA*, *CDKN2A* and *CKDN2B* and *ATRX* loss [25]. More than 50% of EHE tumours have secondary genomic variants, the presence of which may indicate more aggressive disease [24]. 

### 2.4. Clinical Behaviour

Notably, EHE has a variable clinical behaviour: patients with either solitary soft tissue or multifocal lung/liver disease may follow a relatively indolent course, whereas those with either pleural or lymph node involvement, regardless of their primary site or pathologic grade, follow a highly aggressive clinical course similar to a high-grade sarcoma [24]. In general, Mehrabi et al. found that the 1-, 3-, and 5-year survival rates for hepatic EHE, regardless of treatment, were 83.4%, 55.7% and 41.1%, respectively [5]. 

Several sets of predictors of clinical outcomes have been proposed. Clinical factors associated with shorter survival include: clinical baseline tumour-related pain (TRP), development of TRP during follow-up, baseline temperature, and development of fatigue during follow-up [26]; multifocality, nodal involvement, lung primary, and distant metastasis [24,27]. A proposed three-tiered risk assessment system using tumour size and histologic atypia (defined as high mitotic rate, tumour grade and coagulative necrosis) to stratify patients into low-risk, intermediate-risk, and high-risk groups has shown 5-year overall survival rates of 100%, 81.8%, and 16.9%, respectively [28]. Most recently, Li et al. have established and internally validated the first EHE nomogram prognostic model [29]. Based on the Surveillance, Epidemiology, and End Results (SEER) database, they recruited 512 EHE patients and calculated overall survival (OS) at 1, 5, and 10 years for all patients as 76.5%, 57.4%, and 48.2%, respectively. The age, tumour stage, degree of tissue differentiation, surgical treatment, chemotherapy, and radiotherapy were independent factors predicting prognosis [29].

### 2.5. Treatment and Management Principles

There is no universally agreed treatment strategy for EHE due to its rarity and lack of comprehensive molecular characterisation. However, a recent consensus strategy by experts and consumers has been published. 

Treatment strategies include liver transplantation (44.8%), surgical resection for isolated tumours (9.4%) [14,30,31,32], and chemotherapy [5]. Although active surveillance has never been formally studied, it is also a common practice in experienced centres, particularly for a favourable prognostic subgroup [24]. Indeed, active surveillance is the preferred upfront approach in asymptomatic patients (level of evidence V, B) by the ESMO and SPAEN (Sarcoma Patient EuroNet) [14,33]. 

Based on analysis of the World Sarcoma Network database, all systemic treatments for sarcoma have limited activity in EHE. Seventy-three patients (33 treated with anthracycline-based regimens, 11 with weekly paclitaxel, 12 with pazopanib, 15 with IFN-α 2b and 27 with other agents) were included, and none showed meaningful activity [34]. Systemic therapy should be reserved for patients with unresectable disease which is symptomatic and progressive. By contrast the experience with targeted therapies (Table 1), especially with VEGF(R) inhibitors and mTOR inhibitors, is slightly more encouraging, with the potential advantage of a more favourable toxicity profile. In particular, VEGF inhibition with bevacizumab is a promising therapy that exploits the vascular nature of EHE and is well tolerated [35,36]. 

There are currently no reported cases of delivering genomically guided therapies against any of the identified molecular targets. However, novel small molecules targeting the Hippo pathway are in early-phase trials (NCT04665206) [37]. The fusions identified in EHE are also thought to lead to activation of the MEK signalling pathway. Based on this, trametinib is under investigation in an ongoing phase II clinical trial (NCT03148275) [38].

**Table 1:** Targeted Therapies in EHE.

Treatment [Reference]	Study Design and Patient Nos.	Study Outcome [CR, PR, SD, PD]
Sirolimus [39]	A case-series analysis within the Italian Rare Cancer Network for 38 EHE patients	4 PR (10.8%) 28 SD (75.7%) 5 PD (13.5%)
Pazopanib [40]	A retrospective analysis; an EORTC of Soft tissue and Bone Sarcoma group of 10 EHE patients	1 (10%) CR 1 (10%) PR 4 (40%) SD 3 (30%) PD 1 (10%) unknown
Bevacizumab [35]	A multicentre, phase II study with 7 EHE patients	2 PR (29%) 4 SD (57%) 1 PD (14%)
[36]	Case series of 4 EHE patients	3 PR to paclitaxel and bevacizumab. 1 SD on bevacizumab
[41]	Case report of one EHE patient	1 PR to capecitabine and bevacizumb for 6 months.
Sorafenib [42]	Phase II study by the French Sarcoma Group of 15 EHE patients	2 PR lasting 2 and 9 months Non-progression rate of 6 patients (46.5%) at 4 months and 5 patients (38.4%) at 6 months.
Lenalidomide [43]	A case report of one EHE patient	SD 39 months On treatment discontinuation, slight progression seen, responded to rechallenge.
Anlotinib [44]	A case report of one EHE patient	SD for more than 2 years
Lenvatinib [45]	A case report of one EHE patient	PR for 6 months bridging liver transplant

## 3. Materials and Methods

### 3.1. Patient Clinical Data, Samples, and Study Approval

Informed consent was obtained from all patients in accordance with the National Statement of Ethical Conduct in Human Research 2007. Subjects included in this analysis provided consent directly or were consented to the WEHI Stafford Fox Rare Cancer Program (WEHI-SFRCP, Melbourne Health Human Research Ethics Committee 2015.300). Clinical follow-up of patient outcome was obtained from the medical record. Additional human ethics approval was obtained from the Walter and Eliza Hall Institute of Medical Research (HREC #10/05 and #G16/02).

### 3.2. Immunohistochemistry 

FFPE tumour samples were sectioned and stained with haematoxylin and eosin (H&E), or the following antibodies: anti-pan-cytokeratin (mouse, AE1/AE3, Dako, North Sydney, NSW, Australia), anti-CD31 (mouse, JC70A, Dako), anti-CD34 (mouse, QBEnd10, Dako), anti-ERG (rabbit, EPR3864, Roche Diagnostics, North Ryde, NSW, Australia), anti-CAMTA1 (rabbit, polyclonal, Novus Biologicals, Noble Park North, VIC, Australia), and anti-TFE3 (rabbit, EPR11591, Abcam, Melbourne, VIC, Australia). H&E and IHC slides were scanned digitally at 20× magnification using the Pannoramic 1000 scanner (3DHISTECH Ltd., Budapest, Hungary). High-definition images were uploaded into CaseCenter (3DHISTECH Ltd.), and images were processed using FIJI image analysis software 2.14.0 [46].

### 3.3. DNA and RNA Sequencing and Analysis

DNA extracted from the FFPE tumour samples of two patients (#162 and #521) was sequenced using the TruSight Oncology 500 Panel. RNA extracted from FFPE tumour samples of five patients (#154, #368, #455, #503 and #521) was sequenced using the TruSight RNA Fusion Panel. Whole exome sequencing (WES) was carried out on DNA extracted from the FFPE tumour sample of one patient (#368), and whole genome sequencing (WGS) was carried out on DNA extracted from one snap-frozen tumour sample (patient #130). These tumour samples were matched with patient whole blood. 

TruSight RNA Fusion Panel data were analysed by aligning the FASTQ files to the human reference assembly (GRCh37) using STAR aligner13 version 2.7.2b. Arriba [47]. Sample-specific read and coverage metrics were assessed using MultiQC [48].

WES data were analysed using a BioNix pipeline [49] which aligned reads to GRCh38 using minimap2 v2.24 [50], filtering to the Agilent SureSelect Clinical Research Exome V2 capture regions. Small variants were called using Octopus v0.7.0 [51], then annotated with SnpEff v4.3 [52] and dbNSFP v4.2a [53]. Copy number was called using FACETS v0.6.1 [54].

WGS data were analysed by aligning to GRCh38 using BWA mem. Small, copy number and structural variants were called using a consensus of at least two of Mutect2 [55], Strelka2 [56] and Vardict [57] and annotated using the Personalised Cancer Genome Reporter (PCGR) [58], PURPLE [59], and MANTA [60] and BreakPointInspector [59], respectively.

See Supplementary Data for further details of sequencing and analysis.

## 4. Results

### 4.1. EHE Cohort

Through the WEHI-SFRCP, we report a cohort of nine EHE cases with comprehensive histologic and molecular profiling. The diagnoses of EHE were made based on the histopathological examination and confirmed by IHC and, whenever possible, molecular profiling. See Table 2 and Table 3 for a summary of the cohort.

### 4.2. Case #368

**Figure 1:**
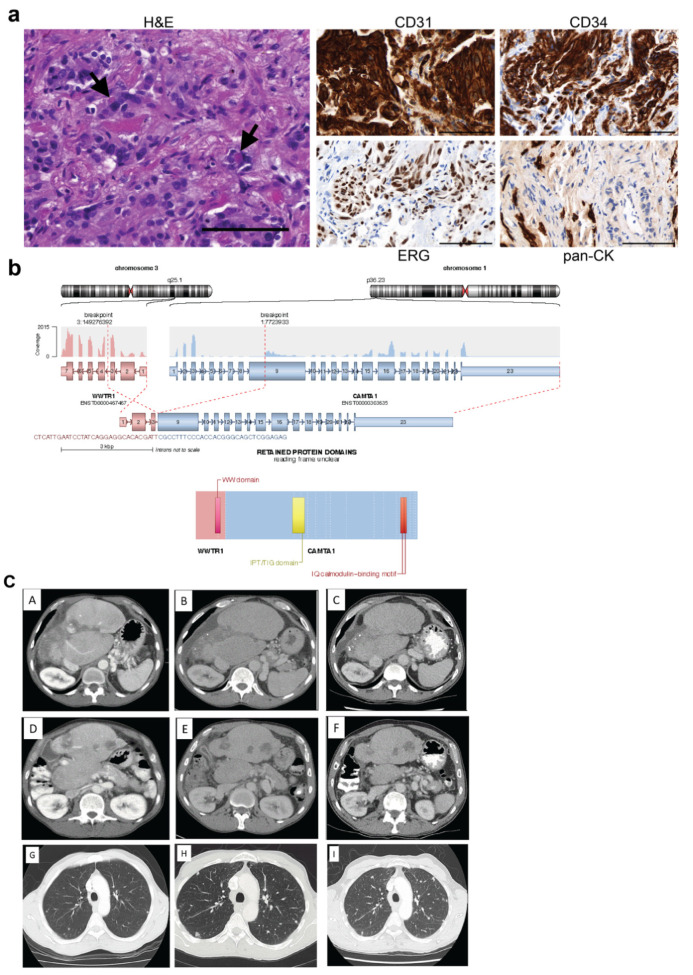
Analysis of case #368. (**a**) FFPE sections from tumour #368 were assessed by IHC. Representative images of H&E, CD31, CD34, ERG and pan-CK staining are shown. Arrows indicate intracytoplasmic red blood cells. Scale bars = 100 μm. (**b**) RNA extracted from tumour sections was analysed using the TruSight Fusion panel. The exons contained within the *WWTR1::CAMTA1* fusion are shown above with the resulting fusion protein shown below. (**c**) CT performed at different time points confirming disease stability. Axial CT images of the liver demonstrating atrophy of the right hepatic lobe, hypertrophy of the left lobe, and multifocal hypodense lesions in 2009 (**A**,**D**), 2013 (**B**,**E**), and 2017 (**C**,**F**). Axial CT images of the lungs showing multiple bilateral small pulmonary nodules in 2009 (**G**) 2013 (**H**), and 2017 (**I**). IHC, immunohistochemistry; H&E, haematoxylin and eosin; pan-CK, pan-cytokeratin; CT, computed tomography.

In October 2003, case #368, a man in his forties, presented with abdominal pain and weight loss (BMI 18) and was found to have deranged liver function tests and multi-focal, hypodense, ill-defined nodules in the right lobe of the liver as well as small bilateral pulmonary metastases on CTCAP. Liver biopsy demonstrated an infiltrating tumour, composed of strands of polygonal epithelioid and spindle cells, set within a fibrous stroma. The tumour cells showed moderate nuclear pleomorphism, hyperchromasia and scanty cytoplasm. Occasional tumour cells showed intra-cytoplasmic lumina that contained red blood cells. IHC revealed strong and diffuse positivity for the endothelial cell markers CD34, CD31 and ERG, while it was negative for the epithelial marker pan-cytokeratin (Figure 1a, Table 3). A diagnosis of EHE was adopted based on its histologic appearance and IHC analysis.

Molecular analysis was carried out on RNA extracted from a later pleural biopsy sample, with the common *WWTR1*::*CAMTA1* fusion identified using the TruSight RNA Fusion Panel. The breakpoints occurred in the third intron of *WWTR1* (location 149,276,392 on chromosome 3) and the ninth exon of *CAMTA1* (location 7,723,933 on chromosome 1) generating a fusion protein containing the WW domain of TAZ and the IPT/TIG domain and IQ calmodulin-binding motif of CAMTA1 (Figure 1b, Table 3 and Supplementary Table S1). Whole exome sequencing (WES) was carried out on DNA extracted from the same sample. Ploidy was found to be 3.7, indicating genome doubling had occurred, and 15% of the genome also displayed loss of heterozygosity (LOH). The tumour had a low tumour mutational burden (TMB). Somatic variants of uncertain significance (VUS) were identified in *ADRBK1*, *SERPINB7, ABCA1, ABCC4* and *ANK1* (predicted to be damaging by in silico predictors but have not been validated) (Table 3).

Due to extensive hepatic disease and extrahepatic involvement, neither radical resection nor liver transplantation was feasible. Review of the literature suggested his disease may follow an indolent course, thus he underwent close clinical surveillance without intervention. 

Two years later, in 2005, he developed progressive right hypochondrial pain with a confirmed radiologic progression. Chemotherapy (carboplatin and etoposide for six cycles) was started. Although the radiologic response was not impressive, he achieved durable pain relief. Subsequently, he developed secondary portal hypertension complicated by oesophageal varices which were effectively controlled by endoscopic banding. Radiological surveillance demonstrated ongoing stable disease (Figure 1c), and he developed atrophy of the right lobe of the liver, mainly segment V and VI, together with hypertrophy of the left lobe. He remains under active surveillance with 6-month clinical review and CT assessment without the need for systemic therapy.

### 4.3. Case #130

**Figure 2:**
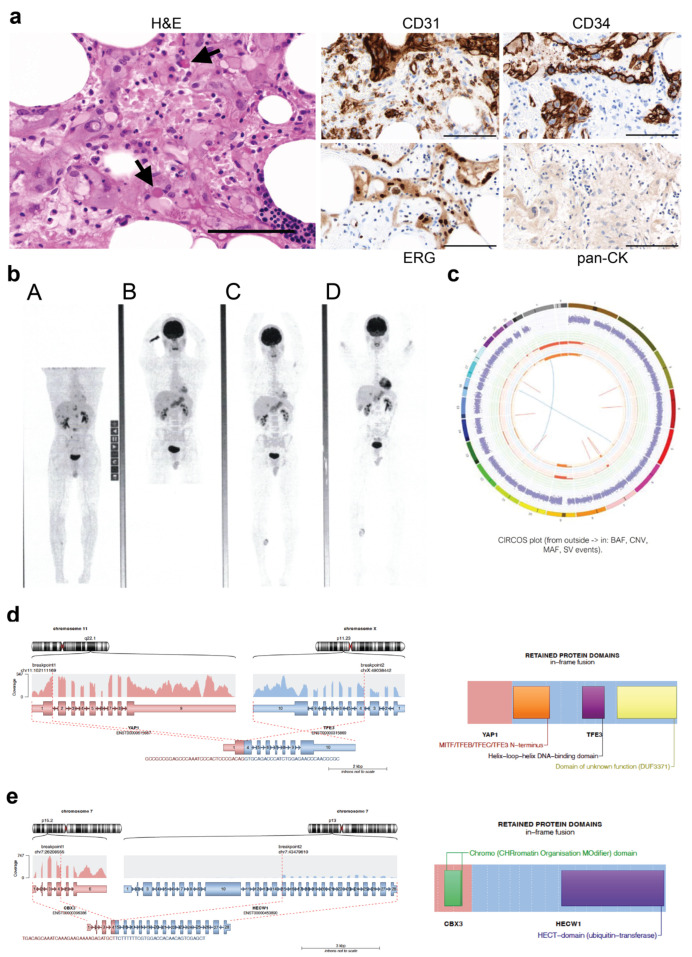
Analysis of case #130. (**a**) FFPE sections from tumour #130 were assessed by IHC. Representative images of H&E, CD31, CD34, ERG and pan-CK staining are shown. Arrows indicate intracytoplasmic red blood cells. Scale bars = 100 μm. (**b**) Whole body PET images in 2010 (**A**), 2015 (**B**), 2017 (**C**), and 2019 (**D**) showing overall stability of the metastatic disease. (**c**) DNA extracted from tumour sections was analysed by whole genome sequencing. CIRCOS plot indicates a tumour with low tumour mutational burden. (**d**) The exons contained within the *YAP1::TFE3* fusion are shown to the left, with the resulting fusion protein shown to the right. (**e**) The exons contained within the *CBX3::HECW1* fusion are shown to the left, with the resulting fusion protein shown to the right. IHC, immunohistochemistry; H&E, haematoxylin and eosin; pan-CK, pan-cytokeratin; PET, positron emission tomography.

Case #130 was diagnosed in 2009 with metastatic EHE. The right medial calf was identified as the primary tumour, with metastatic deposits in the spine, lungs, liver and right humerus. Pathological review indicated the tumour comprised large epithelioid eosinophilic cells, some of which had intracytoplasmic vacuoles. In some areas, the cells were clustered around blood vessels, and some intracytoplasmic red blood cells were noted (Figure 2a). Accompanying fat was infiltrated by tumour and showed mild patchy chronic inflammatory infiltrate. IHC analysis indicated the tumour cells were positive for CD31, CD34 and ERG and negative for pan-cytokeratin (Figure 2a, Table 3). Surveillance positron emission tomography (PET) scans continue to show overall stability of the metastatic disease (Figure 2b). 

Whole genome sequencing (WGS) was carried out on DNA extracted from the primary tumour, indicating a stable genome with very low TMB (0.12 mutations/Mb) with very few chromosomal rearrangements (Figure 2c). No pathogenic variants were identified; however, the less common *YAP1*::*TFE3* fusion was identified (Figure 2d, Table 3). The breakpoints occurred in intron 1 of *YAP1* (location 101,983,686 on chromosome 11) and exon 3 of *TFE3* (location 48,896,661 on the X chromosome) so that a fusion protein containing half of the TEAD-interaction domain of YAP1 and the activation domain (AD), basic helix-loop-helix (bHLH) and leucine zipper (LZ) DNA-binding domains of TFE3 was generated (Figure 2d). Interestingly, a fusion not previously reported in EHE was also identified in case #130, with high confidence and more supporting reads than for the *YAP1*::*TFE3* fusion. This fusion gene was likely to be a passenger. This novel gene fusion (*CBX3::HECW1*) would give rise to a fusion protein containing the Chromatin Organisation Modifier (Chromo) domain of CBX3 and the ubiquitin transferase (HECT) domain of HECW1 (Figure 2d, Table 3).

These two cases exemplify the potential for EHE to follow an indolent course, with both having been followed closely for over 10 years without the need for subsequent intervention. 

### 4.4. Additional EHE Cases in the SFRCP

**Figure 3:**
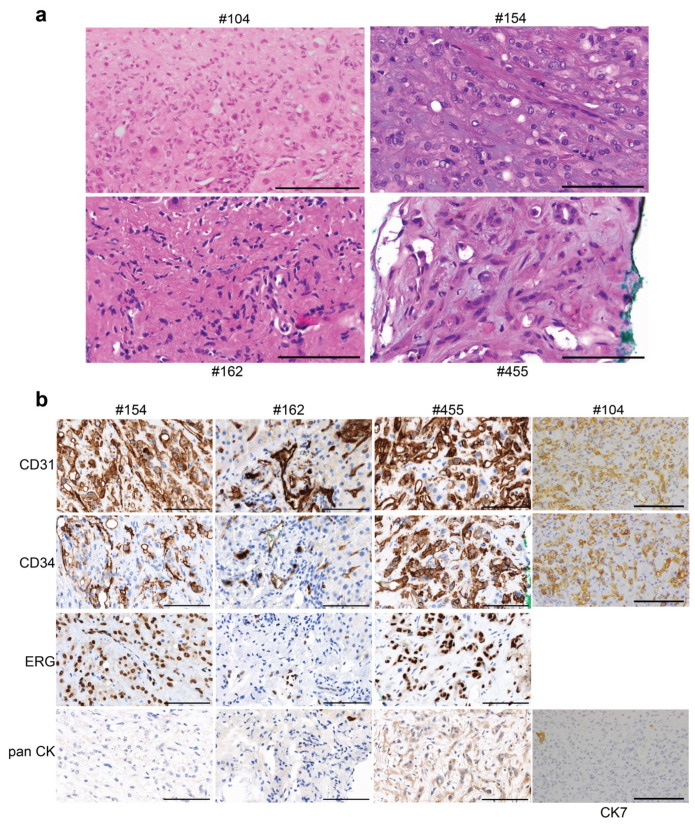
Histological analysis of cases #104, #154, #162, #455, #503 and #521. (**a**) FFPE sections from tumours were stained with H&E for pathological assessment. Scale bars = 100 μm. (**b**) FFPE sections were assessed by IHC. Representative images of H&E, CD31, CD34, ERG and pan-CK staining are shown for five cases. For case #104, CD31, CD34, and CK7 staining was carried out. Scale bars = 100 μm. H&E, haematoxylin and eosin; IHC, immunohistochemistry; pan-CK, pan-cytokeratin.

Six subsequent cases of EHE, #104, #154, #162, #455, #503 and #521, were pathologically assessed. A range of key pathologic features were observed, including plump epithelioid cells and/or spindle cells with enlarged irregular hyperchromatic nuclei, a dense fibrous stroma, and in some cases, apparent intravascular growth and associated mixed inflammatory infiltrate. The cellularity was higher at the periphery of the lesion, with a relatively pauci-cellular and hyalinised central zone. Scattered stromal calcifications were present (Figure 3a). The immunophenotype in all cases was positive for the typical endothelial cell markers CD31, ERG, and CD34 and negative for pan-cytokeratin (Figure 3b, Table 3).

**Figure 4:**
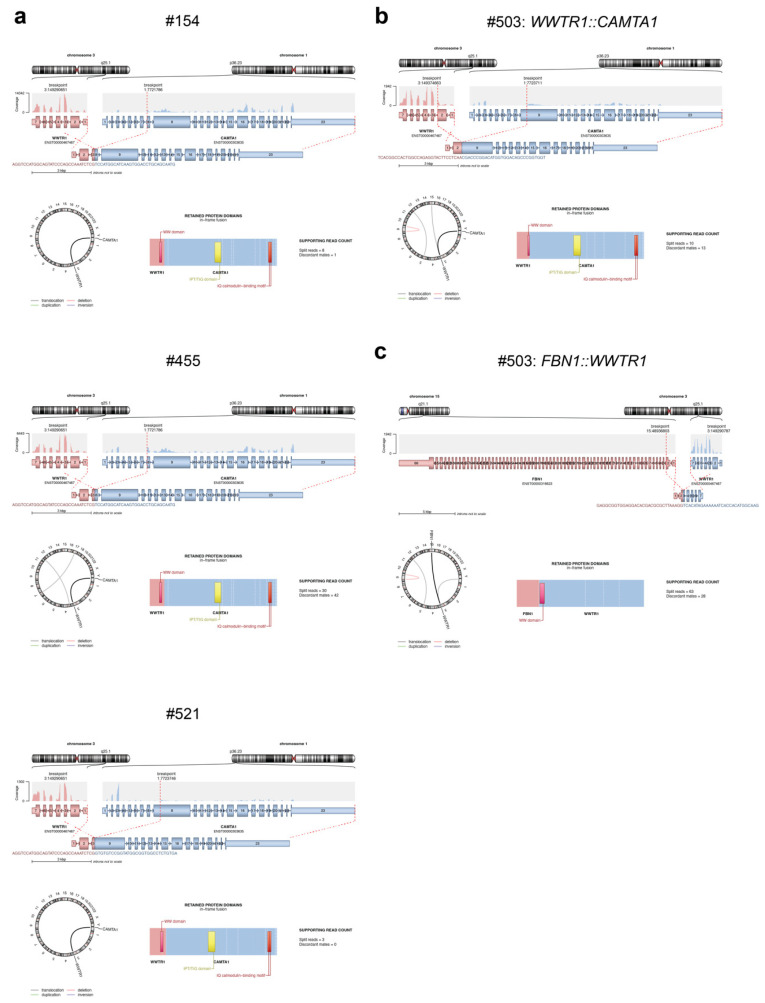
Molecular analysis of cases #154, #455, #521 and #503. RNA extracted from tumour sections was analysed using the TruSight Fusion panel. (**a**) The exons contained within the *WWTR1::CAMTA1* fusions identified in cases #154, #455 and #521 are shown above with the resulting fusion protein shown below for each case. (**b**) The exons contained within the *WWTR1::CAMTA1* fusion identified in case #503 are shown above with the resulting fusion protein shown below. (**c**) The exons contained within the *FBN1::WWTR1* fusion identified in case #503 are shown above with the resulting fusion protein shown below.

The *WWTR1*::*CAMTA1* fusion was identified using RNA extracted from a pleural biopsy for #154, liver biopsy for #455 and liver lesions for #503 and #521 (Figure 4a,b, Table 3). These gene fusions would give rise to fusion proteins containing the WW domain of TAZ and the IPT/TIG domain and IQ calmodulin-binding motif of CAMTA1 (Figure 4a,b, Supplementary Table S1). 

Another fusion not previously reported in EHE was also identified in case #503, with high confidence and more supporting reads than for the *WWTR1::CAMTA1* fusion (Table 3). This novel gene fusion (*FBN1::WWTR1*) would give rise to a fusion protein containing the WW domain of TAZ only, and its function is unknown (Figure 4c, Supplementary Table S1). This case shared pathological features of cases #368, #154, #455, #503 and #521, which also carried the *WWTR1::CAMTA1* fusion, but uniquely demonstrated extension through sinusoidal spaces with atrophy of the intervening liver trabeculae.

DNA extracted from liver tumour samples for two of these six cases, #162 and #521, was analysed using the TruSight Oncology 500 (TSO500) Assay. Both cases had low TMBs, and no pathogenic variants were detected. A VUS in *FBXW7* was identified at an allele frequency of 45.4% in #521 (Table 3). Analysis of RNA extracted from #162 was unsuccessful, and there was insufficient tumour material for #104 to enable molecular analysis to be carried out.

**Figure 5:**
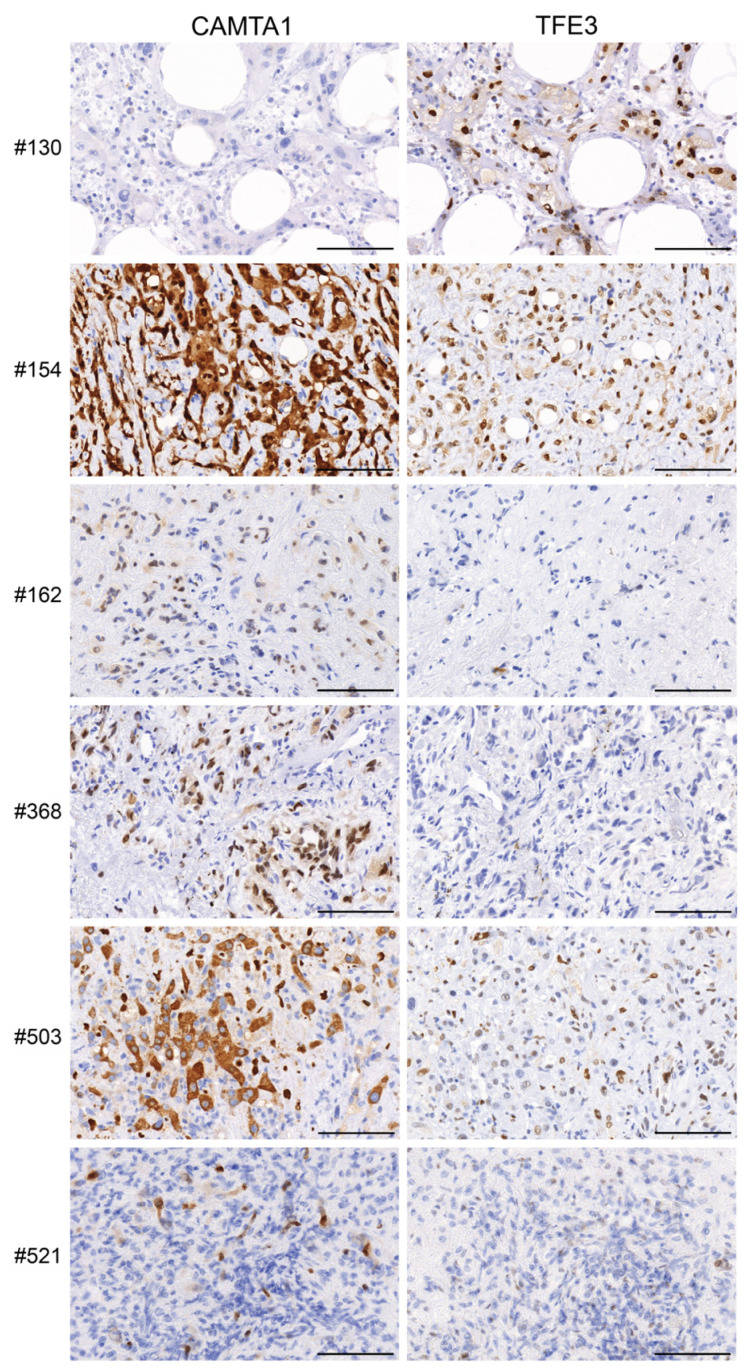
Histological analysis of CAMTA1 and TFE3 expression in cases #130, #154, #162, #368, #503 and #521. FFPE sections from tumours were stained with CAMTA1 and TFE3 antibodies. Representative images are shown. Scale bars = 100 μm.

IHC for CAMTA1 and TFE3 indicated positive staining where expected, with respect to the fusion genes (Figure 5). For example, cases #503, #521, #154, #162 and #368, which all harboured a *WWTR1::CAMTA1* fusion, exhibited positive CAMTA1 staining. The only case harbouring a *YAP1::TFE3* fusion (#130) exhibited positive TFE3 staining. Interestingly, case #154, which did not harbour a rearrangement of TFE3, also exhibited positive TFE3 staining (Figure 5, Table 3). Results are summarised in Table 3. 

## 5. Discussion

A review of the literature for EHE between 1984 and 2022 in PubMed, using EHE as a search criterion, revealed approximately 1800 reviews, case reports and retrospective studies. The results that discussed EHE mimics were considered irrelevant and were excluded from this paper [4,5,31,32,61,62,63,64,65,66,67,68,69,70]. We have reported on the first Australian cohort of nine patients with EHE and delineate histologic and molecular features, clinical details and long-term follow up. 

Molecular analysis in EHE is quite limited in numbers [9,24], and our matched clinical and molecular data provide an important addition to this knowledge base. The *WWTR1::CAMTA1* fusion was identified in five of the six cases where RNA fusion panel sequencing or WGS was possible. This fusion frequency is consistent with others [71,72]. Errani et al. identified that this recurrent translocation was a consistent abnormality in all 17 EHE cases but not detected in any of the morphologic mimics of EHE, such as epithelioid haemangioma or epithelioid angiosarcoma [71]. Furthermore, in another study, *WWTR1::CAMTA1* fusions were detected in 4/7 low-grade and 23/23 intermediate-grade EHE and again were absent in other EHE mimics [72]. Interestingly, Tanas et al., through deep transcriptome sequencing, reported that 87% to 89% of EHE tumours harboured the t(1;3)(q36;q25) translocation, while none of the other 118 EHE mimics showed that rearrangement [73].

The *YAP1::TFE3* fusion was identified in one case (#130; Figure 2d), and this tumour demonstrated unique morphology compared to tumours harbouring the more common *WWTR1::CAMTA1* fusion. This was in concordance with retrospective data from the USA, Italy, and World Sarcoma Network [34,74]. *FBN1::WWTR1* and *CBX3::HECW1*, two novel fusions not previously reported in EHE, were identified in one case each and co-occurred with the *WWTR1::CAMTA1* and *YAP1::TFE3* fusions, respectively. While both novel fusions had more supporting reads than the pathognomonic fusions, they are likely to be passengers, and the function of the resulting fusion proteins is unknown. No known pathogenic gene variants or copy number changes were identified in the four out of nine cases analysed by WGS, WES or TSO500 panel screening. Case #521 had a VUS identified with an allele frequency of 45% in the gene encoding the F-box and WD repeat domain containing 7 (FBXW7) components of the SCF E3 ubiquitin ligase. FBXW7 is a critical tumour suppressor that is frequently mutated in human cancers [75]. Importantly, low expression of FBXW7 is correlated with invasion, metastasis and poor prognosis in a number of cancers [76]. If this variant is validated as pathogenic, this could explain the more aggressive nature of the tumour observed in this case (#521). Case #368 had a variant identified with a frequency of almost 100% in the *ADRBK1* gene, which encodes the G-protein-couple receptor kinase 2 (GRK2). Altered GRK2 expression has been observed in many human cancers and may play a role in angiogenesis [77]. Of particular interest, down-regulation of GRK2 levels in endothelial cells has been found to promote tumour growth [78]. This variant is not classified as pathogenic but is predicted to be damaging, using in silico predictors. While both cases #521 and #368 also had the common EHE fusion gene *WWTR1::CAMTA1*, these additional molecular features may indicate a more aggressive tumour phenotype. Indeed, the presence of secondary genomic variants has previously been proposed to indicate more aggressive EHE [25].

The immunophenotype in all cases was consistent with the known character of this vascular tumour, displaying positivity for the typical endothelial cell markers CD31, CD34 and ERG. In addition, tumours displayed CAMTA1 positivity if they harboured a CAMTA1 fusion and TFE3 positivity in the one tumour harbouring a TFE3 fusion. The tumour for case #154 also displayed positive TFE3 staining despite lacking a *TFE3* rearrangement. This has been observed previously, indicating that TFE3 staining may occur irrespective of TFE3 rearrangement [11,79]. In our cohort, we were not able to associate variation in natural history of this disease with the underlying molecular fusions, although the single case with a *YAP1::TFE3* fusion did appear to have relatively indolent disease compared to most of the cases with *WWTR1::CAMTA1* fusions.

In our cohort, case #368 reported clinically significant symptomatic pain relief after combination carboplatin and etoposide chemotherapy. Pinet et al. reported a complete response to the same regimen in a patient with metastatic pleural EHE disease [80]. By contrast, liposomal doxorubicin [81,82], interferon α [83,84] and thalidomide [85] have shown limited clinical and radiologic responses. Notably, #368 survived 17 years with minimal symptoms, which could have been predicted by some recent nomograms. This patient’s disease displayed few of the many known poor prognostic features (TRP and weight loss). Applying the prognostic nomogram—in retrospect—predicted a 30% chance of survival at 200 months [86]. This reinforces that proposed predictive models of an indolent course should be validated by international aggregation of cases to better predict patient outcome and avoid the toxicity of systemic therapy, which often fails to achieve disease response. 

## 6. Conclusions

EHE is an ultra-rare, vascular tumour that often presents incidentally and has an unpredictable clinical course. Histologically, it can be mistaken for other benign and malignant vascular-epithelial tumours. However, it can be differentiated by IHC staining for CD31, CD34 and ERG, as well as by molecular testing. Our analysis of nine EHE cases harbouring the two gene-defining molecular abnormalities, *WWTR1::CAMTA1* and *YAP1::TFE3*, supports previous reports that these abnormalities define different biologic entities. This group exemplifies the need to characterise the natural history of rare cancers, as well as identifying underlying genomic drivers. Advancing therapeutic progress for rare tumours requires a precision medicine approach using genomic sequencing and basket trial designs. In the case of EHE, a watch-and-wait approach should be considered, to spare patients with indolent disease from potentially unnecessary treatments. Further work is warranted to determine if the *YAP1::TFE3* fusion predicts a less aggressive course than the *WWTR1::CAMTA1* fusion. 

Analysis of this cohort of EHE was achieved through the WEHI-SFRCP, facilitated through enrolment in the ARC Portal, providing matched clinical, molecular, and histopathological characterisation of rare cancers in the hope of improving treatment options. Cohort analyses such as this, achieved through national collaboration, are critical to advancing our knowledge of ultra-rare cancers. 

### Limitations of the Study

An obvious limitation of any study involving a rare cancer will always be cohort size. Considering the incidence of EHE is 1 per 1,000,000 population, our cohort of nine patients is quite impressive. While a small cohort size makes it difficult to draw significant associations, it is extremely important to make the results widely available so they can be added to the already presented data on this rare cancer. By combining the results of many small studies, we are able to advance our knowledge of this ultra-rare cancer.

## Figures and Tables

**Table 2:** Clinical summary of EHE cohort.

Case (Age at Diagnosis and Sex)	Diagnosis	Treatment	Current Clinical Course (Censored at the Time of Data Collection)
#104 (60s F)	Hepatic EHE, pulmonary and T12 bony metastases	Weekly paclitaxel completed with radiological SD, but improvement in pain burden. Upon progression, rechallenge weekly paclitaxel with poor tolerance	8 years active surveillance
#130 (40s M)	Right calf extrahepatic EHE with metastasis to spine, pulmonary, hepatic, and right humerus	10 years of surveillance Upon progression, definitive preoperative radiotherapy with surgical resection of calf primary	13 years active surveillance
#154 (50s M)	Pleural EHE Progressive symptomatic pleural effusion and new bony lesions in right ilium, left clavicle and left sacrum	Surgical pleurodesis for pleural effusion	6 years active surveillance
#162 (60s F)	Hepatic EHE and small volume pulmonary nodules	Resection of liver lesion with clear margins	5 years active surveillance
#368 (60s M)	Hepatic EHE and small volume pulmonary nodules	Carboplatin/Etoposide without radiological response	20 years active surveillance
#455 (70s M)	Hepatic EHE	Active surveillance only	21 years active surveillance
#499 (60s F)	Extrahepatic EHE of the left popliteal fossa with pulmonary metastases	Radiotherapy to left knee Radiotherapy 56 Gy to left inguinal nodal disease	2 years of active surveillance—lost to follow-up
#503 (30s F)	Hepatic EHE	Inoperable, awaiting liver transplant	2 years of follow-up
#521 (40s F)	Hepatic EHE and pulmonary metastases	Liver resection	2 years of follow-up

CT, computerised tomography. F, female, FDG-PET, fluorodeoxyglucose positron emission tomography, M, male.

**Table 3:** Histopathological and molecular summary of EHE cohort.

Case	Molecular Analysis Completed	Molecular Data	Histopathology IHC (Positive)
#104	N/A	N/A	CD31, CD34, CD10
#130	WGS	*CBX3::HECW1* and *YAP1::TFE3* fusions	CD31, CD34, ERG, TFE3
#154	TSF	*WWTR1::CAMTA1* fusion	CD31, CD34, ERG, CAMTA1, TFE3, retained BAP1
#162	TSO500	Low TMB No clinically significant variants	CD31, CD34, ERG. CAMTA1
#368	TSF, WES	*WWTR1::CAMTA1* fusion No pathogenic variants; somatic VUSs: *SERPINB7, ABCA1, ABCC4, ANK1* and *FOXK1*	CD31, CD34, ERG, CAMTA1
#455	TSF	*WWTR1::CAMTA1* fusion	CD31, CD34, ERG, CAMTA1
#499	N/A	N/A	CD31, CD34, ERG, SMA, S100, CK8/18
#503	TSF	*WWTR1::CAMTA1* and *FBN1::WWTR1* fusions	ERG, CD31, CD34, CAMTA1, TFE3 (a few cells)
#521	TSF, TSO500	*WWTR1::CAMTA1* fusion Low TMB No clinically significant variants Somatic VUS in *FBXW7*	ERG, CD31, CD34, CAMTA1

N/A, not available; WGS, whole genome sequencing; TSF, TruSight Fusion panel; TSO500, TruSight Oncology 500 panel; TMB, tumour mutational burden; WES, whole exome sequencing; VUS, variant of uncertain significance.

## Data Availability

Sequence data for cases #130 and #368 have been deposited at the European Genome-phenome Archive (EGA), which is hosted by the EBI and the CRG, under accession number EGAS00001007474. Further information about EGA can be found on https://ega-archive.org (accessed on 7 August 2023). The European Genome-phenome Archive of human data consented for biomedical research”. A data transfer agreement is required.

## Supplementary Materials

The following supporting information can be downloaded at: https://www.mdpi.com/article/10.3390/cancers15174378/s1, Supplementary Table S1: Details of fusion genes identified using the TruSight Fusion Panel or WGS, Supplementary methods [49,50,51,52,53,54,59,60,87,88,89,90,91,92,93,94].
