# Supplementary material for: A Matched Molecular and Clinical Analysis of the Epithelioid Haemangioendothelioma Cohort in the Stafford Fox Rare Cancer Program and Contextual Literature Review
Source: Cancers (Basel). 2023 Sep 1;15(17):4378. doi: 10.3390/cancers15174378 (PMC10487006; doi:10.3390/cancers15174378)
Supplement: Supplementary file 1 [file cancers-15-04378-s001.zip › cancers-2553438-supplementary - xml - Copy.pdf]

Supplementary Methods

DNA and RNA sequencing

TruSight RNA Fusion Panel analysis was carried out on RNA was extracted from formalin-fixed, paraffin-embedded (FFPE) using the AllPrep DNA/RNA kit (Qiagen) with semi-automation using the QiaCube Connect according to the manufacturer's protocol. 100 ng RNA, as determined by Qubit (ThermoFisher Scientific), was used as input. RNA libraries were prepared using the TruSight RNA Fusion Panel library preparation kit following the manufacturer's instructions. Briefly, double-stranded, blunt end cDNA was generated from FFPE-derived RNA. Following A-tailing and the ligation of sample-specific adaptors a pre-capture amplification was performed and library quality and quantity was assessed using TapeStation (Agilent). Between 50 and 200 ng of pre-capture library was subjected to two rounds of hybridisation using probes directed at 507 tumour-associated fusion genes, followed by streptavidin bead-based capture and washing. Following the second round of capture/washes the libraries underwent a final round of PCR amplification followed by bead-based (Ampure, Beckman Coulter) cleanup and a final quality and quantity assessment using TapeStation (Agilent) analysis. Equimolar quantities of between 6 and 10 samples were sequenced on a MiSeq v3 system (Illumina) using 2 × 75 bp paired-end reads.

Whole exome sequencing (WES) of patient #368 was performed on DNA extracted from FFPE tumour tissue and matched blood. 150–300 ng of DNA was fragmented to approximately 200 bp using a focal acoustic device (Covaris S2, Sage Sciences). Libraries were prepared with the Kapa Hyper Prep Kit (Kapa Biosystems) and SureSelectXT adaptors (Agilent). Hybridisation capture was performed with SureSelect Clinical Research Exome V2 baits following the SureSelectXT recommended protocol (Agilent). Indexed libraries were sequenced on an Illumina NovaSeq 6000 (Illumina) to generate paired-end 150 bp reads with average of 70-fold base coverage for the germline (blood) sample and 330-fold coverage for tumour sample.

Whole genome sequencing (WGS) was carried out on DNA extracted from one snap frozen tumour sample (#130) and matched whole blood using the QIAamp DNA Mini Kit (Qiagen). DNA was quantified using the Qubit dsDNA HS or BR kits (ThermoFisher Scientific). Libraries were prepared using the Illumina TruSeq Nano library method using 200ng of DNA. Extracted DNA was sheared using the Covaris M220 Focused-ultrasonicator with a target fragment length of 550bp through bead size selection. The Illumina TruSeq nano DNA library preparation kit was

used for End repair and Adenylation of 3’ fragment end. The adaptor was ligated, then the molecular barcode/index was incorporated through amplification. The libraries were assessed for quality (Qubit, TapeStation4200 and KAPA Illumina library quantification kit using qPCR QuantStudio6) prior to normalisation and pooling before loading onto the Illumina NovaSeq 6000 for sequencing at depth of 40x for germline DNA and 80-100x for tumour DNA using paired 150bp reads. Sequencing on the NovaSeq was performed following Illumina’s Sequencing by Synthesis technology. The denatured single strand library were loaded into a flow cell where fragments were captured on a lawn of surface bound oligos inside patterned microwells. Upon hybridisation amplification began immediately. Each fragment was then amplified into distinct clonal clusters through ExAmp Cluster generation. A fluorescently labelled reversible terminator was imaged as each dNTP was added then cleaved to allow incorporation of the next base. Each image was converted to a BCL file by Illumina software RTA3. Each BCL file was demultiplexed and converted to a FASTQ file using Illumina BCL Convert 3.9.3 for further analysis including alignment and annotation.

Genome sequencing analysis

WES of patient samples was analysed using a BioNix [49] pipeline to process samples from sequencing data to variant calls. Sequences were aligned to Genome Reference Consortium Human Build 38 (GRCh38) using minimap2 v2.17 [50]. WES used the Agilent SureSelect Clinical Research Exome V2, with reads filtered to 100bp each side of capture regions. Small mutations were called using Octopus v0.7.0 [51] and annotated using SnpEff v4.3 [52]. Copy number variants were called using FACETS v0.6.1 [54]. Structural variants were called using GRIDSS (v2.13.2) [87,88] and annotated using StructuralVariantAnnotation (Version 1.12.0) [89]. Single nucleotide variants were checked against the ClinVar public archive of reports of the relationships among human variations and phenotypes (https://www.ncbi.nlm.nih.gov/clinvar/), and in-silico predictor of pathogenicity MutationTaster [90], using dbNSFP v4 [53].

WGS analysis was performed using a pipeline developed in the University of Melbourne Centre for Cancer Research as follows (hereafter referred to as the UoM pipeline). Sequence reads were aligned to the GRch38 build of the human reference genome using BWA mem. Variants were detected by at least 2 of the following mutation callers (Mutect2, Strelka2 & Vardict) using the BCBIO pipeline (https://github.com/chapmanb/bcbio-nextgen). All variants were annotated using

the personalised cancer genome reporter (https://github.com/sigven/pcgr). Single Nucleotide Variants (SNV)/Indels were classified according to a five-tiered structure, similar to proposed recommendations [91], also adopting the MLVD framework for description of clinically relevant cancer variants. Tumour mutational burden (TMB) was defined as the number of coding, somatic substitutions and indels, including synonymous alterations, per megabase of the targeted coding genomic region (34MB) [92]. Copy number variants were called using PURPLE [59]. Structural variants were detected using MANTA [60] and BreakPointInspector [59]. CNV and SV changes are annotated with the *svprioritize* (https://github.com/AstraZeneca-NGS/simple_sv_annotation) framework assigning priority to fusion events, whole exon loss or upstream/downstream changes for a list of 1246 cancer-associated genes only (https://github.com/umccr/workflows/blob/master/genes/cancer_genes/umccr_cancer_genes.latest.ts). Somatic mutations are assigned to COSMIC v2 mutational signatures [93] using the MutationalPatterns framework [94].
